# Chloride of Magnesium

**Published:** 1844-07-27

**Authors:** 


### CHLORIDE OF MAGNESIUM.

This has lately been recommended as a saline aperient by Dr. Lebert. It is said to produce no injurious effect whatever on the stomach, and if occasionally it gives rise to any unpleasant sensation, it inconveniences less than most other purgatives. It would seem to favour digestion, since its purgative action is followed by an improvement of the appetite. The mean dose as an aperient is 30 grammes for an adult, and half that quantity for a child of from ten to fourteen years of age.—*Prov. Med. and Sur. Journ.* from *Gaz. Med. de Paris.*

